# Entrepreneurship, beekeeping, and health training to decrease community violence in Dar es Salaam, Tanzania: a pilot study for an intervention trial

**DOI:** 10.1186/s40814-021-00920-1

**Published:** 2021-10-04

**Authors:** Anne H. Outwater, Alison G. Abraham, Masunga K. Iseselo, Linda Helgesson Sekei, Method R. Kazaura, Japheth Killewo

**Affiliations:** 1 Department of Community Health Nursing, School of Nursing, Muhimbili University of Health and Allied Sciences, Dar es Salaam, United Republic of Tanzania; 2 Department of Epidemiology, School of Public Health, University of Colorado, Denver, CO USA; 3 Department of Clinical Nursing, School of Nursing, Muhimbili University of Health and Allied Sciences, Dar es Salaam, United Republic of Tanzania; 4 NIRAS International Consulting, Copenhagen, Denmark; 5 Department of Epidemiology and Biostatistics, School of Public Health and Social Sciences, Muhimbili University of Health and Allied Sciences, Dar es Salaam, United Republic of Tanzania

**Keywords:** Microbusiness, Informal sector, Africa, Entrepreneurship training, Youth unemployment, Violence reduction, Beekeeping, Informal economy, Microfinance, Urban youth

## Abstract

**Background:**

High unemployment rates and limited access to resources, services, and economic opportunities are associated with many types of violence. In Dar es Salaam, Tanzania, most violence is experienced by unemployed, poorly educated men between the ages of 20 and 35 years. It is expected that community violence will decrease as the incomes of those most at risk increase. However, economic opportunity through formal employment is rarely available to uneducated men in Dar es Salaam. Giving them access to economic independence through entrepreneurship training is therefore supported by the World Bank and the government of Tanzania. There has been little research on the effectiveness of programs to encourage entrepreneurship.

**Methods:**

To evaluate the feasibility of providing entrepreneurial training programs to young men in Dar es Salaam, especially those without formal employment, a pretest-posttest pilot study was conducted drawing a sample of young men from neighborhood camps called *vijiweni.* There were four interventions, each implemented in a single camp: Health/Control, Entrepreneurship + Health, Beekeeping + Health, and Entrepreneurship + Beekeeping + Health. The four camps received 2, 6, 6, and 10 training sessions, respectively. No start-up capital was provided. The participants were interviewed at baseline and 3 months, 6 months, and 1 year after the sessions were completed. Data were collected on demographics, household assets, experience of violence, and income.

**Results:**

Fifty-seven respondents attended the first session. At baseline, the camps were not meaningfully different from one another in educational attainment, number of dependents, daily income, assets, or individual members’ roles as victims, perpetrators, or witnesses of violence. Differences were found in age, occupation, and weekly income. Over a period of 2.25 years (from baseline to the end of the project), the weekly income of the Health/Control camp, which had been earning the most, decreased by 37% in a reflection of worsening economic conditions at the time. All three intervention camps increased their income: Beekeeping by 43%, All by 50%, and Entrepreneurship by 146%, with the latter almost reaching the minimum wage level. The most persistently reported constraint was insufficient start-up capital.

**Conclusions:**

The feasibility and potential effectiveness of a short training program on entrepreneurship skills for unemployed, poorly educated young men in urban Tanzania were demonstrated in this study. It has set the stage for an intervention trial to test an updated hypothesis: A 5–7-day intervention about entrepreneurship and microfinance savings groups will lead to increased income and decreased violence.

**Trial registration:**

ClinicalTrials.govNCT 04602416. Registered on 24 October 2020. Retrospectively registered.

## Key messages regarding feasibility 

What uncertainties existed regarding the feasibility of the study?

- Could the target group be found at the camps (*vijiweni*)?
- Would the target group members attend sessions regularly and attentively? Act appropriately on campus and during field and classroom sessions?
- Was the curriculum appropriate to their level of understanding?
- How much honey would be produced?
- How much money would the participants earn?
- Could their incomes be increased to reach a basic standard of living?

What are the key feasibility findings?

- The target group can be found at the camps.
- The target population was willing to be engaged.
- All four interventions were feasible to implement.
- Beekeeping production can be delayed by factors beyond the beekeepers’ control.
- We have parameters for sample size calculations for a future cluster randomized trial.

What are the implications of the feasibility findings for the design of the main study?

- A short training program on entrepreneurship skills for underemployed, poorly educated young men in Dar es Salaam is feasible and potentially effective.
- Beekeeping can provide a source of supplementary income for rural youth, in that the hives can be situated near their homes in natural pesticide-free areas.
- This study has set the stage to test a hypothesis: An intervention lasting 5–7-days on entrepreneurship and/or microfinance will lead to increased earnings, decreased experiences of violence, and greater overall well-being.

## Background

Most violence in Dar es Salaam, a city of about 6.7 million people in the East African country of the United Republic of Tanzania, is committed by and against young men [29]. The most significant predictors of homicide death are unemployment, poor education, and living alone [18]. Young, underemployed men with poor educations are also at risk of recruitment into non-national armies such as the militant Islamist group al-Shabaab [30]. However, such young men have the potential to contribute to national economic empowerment if they receive the right training and support.

Based on theory [9] and previous research (e.g., [2, 30]), it is hypothesized that community violence will decrease as the incomes of those most at risk increase. Intervention around employment status is the most feasible way of achieving this objective. Most occupations open to uneducated young men are inherently unstable, as they are generally seasonal, short-term, or illegal [4, 30]. In national economies in which formal jobs are in short supply, job creation is difficult, and there is a large youth population to absorb. Most African governments and multinational financial institutions agree that entrepreneurship is an important option for securing economic and political security [35, 38].

Self-employment and entrepreneurship are encouraged by the Tanzanian government. Several initiatives have emerged in Tanzania to address youth unemployment. Nongovernmental organizations (NGOs), including the International Labor Organization (ILO), Plan International, Restless Development, TechnoServe, Femina HIP, and others, have started implementing projects to address the lack of opportunities for productive economic engagement by youth. Novel strategies include the establishment of beekeeping enterprises in youth groups supported by the Tanzania Forest Service. Little research data are available on the effectiveness of such initiatives even though they have the potential for positive results.

In East Africa, 91.6% of people over 15 years old are employed: 83% informally, 4.8% formally, and 3.8% in households [16]. A slightly greater proportion of youth ages 15–24 years, 96.7%, is employed in the informal sector. Employment in the informal sector is most common in trade-related activities, including entrepreneurial street vending. Education levels are closely linked to informal employment in Africa. Those with no education tend to be in highly informal work (94.0%). The rate of informality falls to 88.5% with primary education and further decreases to 68.1% for those with a secondary education and to 27.0% for those with a tertiary education. It is to be expected that our low-income, poorly educated participants would be among those active in the informal economic sector.

Our study participants were part of the 60% of the world’s employed population who earn their livelihoods in the informal economy [16]. Among all the regions of the world, the highest proportion of youth entrepreneurs is to be found in the Africa Region [8]. One-third of these young people are necessity-driven entrepreneurs, which means that they perceive entrepreneurship as a survival strategy and not as a business opportunity. Few studies have examined entrepreneurial and microenterprise programs with a focus on young men at risk for violence, especially in Africa. Most research on entrepreneurial and microenterprise programs in low-income countries centers on the issue of HIV/AIDS, with a particular focus on women, who traditionally have not been income earners [10]. Besides focusing on a different population, such interventions are often poorly described, and programmatic content is routinely omitted. Studies are typically single-group postintervention assessments using quantitative, qualitative, or mixed methods. Most are also flawed by small sample sizes and a lack of controls or objective longitudinal measures [10]. Thus, there has been a dearth of rigorous research on entrepreneurial and microenterprise programs.

Smith and Perks [33] postulated that the main factor in sustaining employment in the informal sector is training in entrepreneurial skills. They found that Black microentrepreneurs in the informal sector in South Africa possessed very limited skills, mainly due to a lack of formal education and training. Using in-depth interviews, Smith and Perks explored which skills were important for successful Black microentrepreneurs in South Africa. The researchers outlined more than three dozen skills needed for four main types of successful entrepreneurial practice: personal, technical, business operations, and management.

Lack of appropriate training and work experience are major obstacles [7, 34]. In Zambia, it was found that formal education may not provide the skills needed for productive entrepreneurship, but business training can [7]. Brixiová et al. [6] showed that in Swaziland a positive relationship existed between the amount of business training young entrepreneurs received and their level of performance—that is, the more training they had received, the more likely they were to be successful. In Ethiopia, entrepreneurial training was shown to be a pull factor stimulating youth to be self-employed [24].

Maman et al. [21] conducted an intervention trial with young men in Dar es Salaam, Tanzania, who underwent 5 days of training on business development, entrepreneurship, and finance led by a local microfinance institute as part of a $100 loan with 18% (6 months) interest and 27% (9 months) interest, then were evaluated 30 months later for primary outcomes, including intimate partner violence perpetration. Quantification of the outcomes was accomplished with an adapted version of the World Health Organization’s Violence Against Woman instrument. The mean age of the participants was 26 years. It was reported that 56.8% of them had a primary school education or less; 11.8% had attended some secondary school; the rest (31.4%) had at least completed secondary school. About a sixth of the men, 16.4%, reported perpetrating one or more incidents of intimate partner violence over the past year. The intervention (including weekly follow-up by loan officers for loan repayment, and booster sessions every 6 months with peer health educators) did not significantly affect reported intimate partner violence perpetration after 30 months. Income change as a possible result of the loan was not reported.

In order to address some of the methodological research gaps [7, 33, 34], an intervention trial with four interventions involving training in entrepreneurship, beekeeping, and health education was envisioned. Pilot/feasibility studies are an important step in any major trial that seeks to evaluate an intervention [15, 20]. In the present article, we report on a pilot study focusing on entrepreneurship, beekeeping, and health training that was conducted to inform such an intervention trial.

The objectives of the pilot study included the following:

1. To evaluate the suitability of informal camps (*vijiweni*) frequented by young men as places to recruit the target group
2. To evaluate the suitability of the curriculum
3. To quantify bee product production after 1 year
4. To gain insight into the income generation potential of four interventions after 3, 6, and 12 months
5. To obtain the required preliminary data for the calculation of a sample size for the primary outcome

## Methods

This study used a pretest-posttest design with four different interventions.

### Site and setting

Tanzania is a low-income country on the east coast of Africa. The fast-growing metropolis of Dar es Salaam, the nation’s commercial hub, is home to almost 10% of the national population—about 6.7 million people. Dar es Salaam has a fairly young population: 33.6% of its residents are between the ages of 20 and 34 years [27]. About 75% of male students finish primary school, about 49% go on to the ordinary level (lower secondary school), and 12% continue to the advanced level (upper secondary school) [11].

### Selection of study population and sample

The target population for the present study was Tanzanian men between the ages of 20 and 35 years, without formal employment. To gain access to this population, we built on the experience of Yamanis et al. [39], who discovered that most young men in Dar es Salaam, especially those without formal employment, joined *vijiweni*, camps that were characterized by a specific geographical location, a unique name, a democratic system of leadership, and membership dues. Yamanis et al. reported that the members of the *vijiweni* displayed strong social bonds, often engaging in group activities such as football and music. The authors concluded that these were appropriate groups for interventions with low-income youth. Iseselo et al. [17] described the process through which four camps out of a possible 71 were chosen for the present study. Participation of the camps was solicited through an invitation to the camp leader. Each leader was told that 15 men from his camp, ages 20–35 years, were welcome to attend an introductory session, to be held on 24 April 2015; they could make their decision whether to join the study then. All invited camps sent members to the introductory session.

### Assignment of camps to study interventions

Our first session with the study participants entailed an introduction to the research project and acquisition of informed consent. After they gave informed consent (which all did), a baseline interview was conducted. After the baseline interviews, we pseudo-randomized the four camps into the four interventions, in a process that was witnessed by the participants. The four interventions, Health/Control, Entrepreneurship, Beekeeping, and All, were respectively assigned the numbers 1–4. Camps were assigned to interventions on the basis of a drawing in which camp leaders drew a number, either 1, 2, 3, or 4, from a matte plastic envelope. Postintervention data collection occurred at intervals of 3, 6, and 12 months after the end of the intervention.

### Description of the interventions

We developed training that was based on knowledge and models currently used in Tanzania; it was not substantively tailored to our project. All camps, including Health/Control, participated in two sessions: Introduction and Mind Your Health. The health sessions were modeled on sessions given to United States Peace Corps Volunteers about important preventable diseases found in Tanzania, including HIV/AIDS, malaria, diarrhea, worms, and malnutrition. The Entrepreneurship intervention was designed by Femina Hip, a Tanzanian civil society organization with a focus on edutainment, and was based on the organization’s experience producing an entrepreneurship competition series, *Ruka Juu!* (“Jump Up!”), that had been aired on national television in 2011 and 2013. The aim of the edutainment series was to teach young people in Tanzania about entrepreneurship and inspire them to venture into small businesses and agriculture [1]. Members of the Entrepreneurship camp were given six sessions, the Health/Control sessions plus four more: Sources of Capital, Saving and Investing for Profit, Business Plan, and Marketing/Customer Care. Videos of the original *Ruka Juu!* series were shown to the group in a classroom; the facilitators for the television programs and our sessions were the same. Coincidentally, during the study, the Entrepreneurship camp received a second dose of entrepreneurship training outside the context of the study. This consisted of an intensive 5-day course offered by an international NGO called Restless Development which was implementing training projects tailored for poorly educated people without capital in Dar es Salaam to build financial literacy and employability, as well as skills in entrepreneurship and business planning. The similarities and differences between the Femina Hip and Restless Development trainings are shown in Table 1.

**Table 1:** Differences between the two entrepreneurship trainings received by the Entrepreneurship camp

Item	Femina Hip	Restless Development
**Format**	Adapted from 5 of 11 television shows in an edutainment series, *Ruku Juu!*	ILO-designed curriculum for entrepreneurship training
**Duration of training**	1 year	5 days
**Days of training**	5	5
**Target group**	Farmers growing market crops	Underemployed, undereducated people
**Training components**	Introduction; Entrepreneurship; Saving/Profits; Customer Service/Marketing; Business Plan	Entrepreneurial Mindset; Marketing; Costs/Pricing; Financing; Business Plan

The beekeeping intervention was designed by officials from the Tanzania Forest Services, an agency of the Ministry of Natural Resources and Tourism. The beekeeping intervention consisted of six sessions: the Health/Controls’ two sessions plus Beginning Beekeeping, Building Beehives, Hive Placement, and Harvesting. The latter three were field sessions. The members of the All intervention camp participated in all 10 sessions. All participants received photocopies of important learning materials and a folder. The Beekeeping and All camps were each provided equipment and materials for building, hanging, and harvesting four hives.

Sessions were delivered one per day by trainers from Femina Hip (entrepreneurship), the Tanzania Forest Services (beekeeping), and the US Peace Corps (health) from April 2015 to July 2016. All sessions took place on the campus of the Muhimbili University of Health and Allied Sciences (MUHAS), in Dar es Salaam, except for three beekeeping field sessions that were presented in Kongowe National Forest, 24 km west of the city. The sessions took place at the time most convenient for the participants, Saturdays from 9 a.m. to 5 p.m.

Although entrepreneurship and start-up capital are often considered together, no intervention included start-up loans or grants to the camps or individuals, for two reasons: (a) giving support of this kind to people or groups with little experience in business might not be sustainable over time in such a setting and (b) receiving such support could cause divisions among the participants in a particular intervention. Individuals in different camps shared information in great detail [17]. If one group were getting money and the others were not, resentment could result. Also, it is possible that the groups without money might give up, thus creating a self-fulfilling prophecy that nothing can be accomplished without capital.

Study participants were given nutritious lunches and tea each time they attended a session. They were also given 13,000 Tanzanian shillings (TzS), the approximate equivalent of US$7, to reimburse them for bus fare and any daily earnings lost due to attendance at the sessions.

### Data collection and covariates

Using the same questionnaire each time, members of our research team interviewed the participants four times: at baseline, then at 3 months, 6 months, and 1 year postintervention. The same 10-member team conducted all interviews. Participants were invited to the MUHAS campus for face-to-face interviews. Those who could not come were contacted by telephone; if they were willing to be interviewed, this was done at their campsite. Since several questions were potentially sensitive, respondents were informed that they should refuse to answer any that made them feel uncomfortable rather than give an untrue answer.

Self-reported data were collected on the following variables: age, education, occupation, number of dependents, assets (by means of an asset index), daily and weekly incomes, and experiences of violence. On the basis of responses to an open-ended question on occupation, data on this variable were sorted into five categories: microbusiness, drivers, unskilled and day laborers, semiskilled workers who had gone through at least a minimum of specialized training, and respondents who were dependent on family members, including full-time students. The asset index was created on the basis of information on whether the respondent owned a bicycle, motorcycle, or cell phone; lived in a house (and whether the house was on a family plot of land and included flooring and a toilet); and had a bed to sleep in.

The definition of income we used was “receipts of an active economic unit (person) over a defined time period” ([36], p. 308). Income is a challenging variable to measure when precise measurement is needed of multiple sources of revenue such as regular, irregular, temporary, and seasonal [36]. Among our study participants, sources of income changed daily, so short reference periods were used to facilitate detailed recollections. Income was explored through open-ended questions: What was your income yesterday? Last week? The questions were read the same way to each respondent. Probing was used to aggregate different revenue streams in the specific time span in order to increase the accuracy of the data.

According to the commonly used World Health Organization typology, interpersonal violence has four modes: physical, sexual, psychological, and deprivation/neglect [19]. Interpersonal violence can be divided into two main types: family/partner and community. Community violence is further divided into that occurring among acquaintances and that among strangers and includes forms of mob violence such as lynching and vigilantism.

Exposures to violence were assessed through two close-ended questions (Have you experienced violence in the community? When?) and an open-ended question (Describe the incident). The open-ended question was used to capture the respondents’ perceptions of violence rather than emphasize the researchers’ preconceived definitions. On the basis of the description of a specific incident, it was possible to categorize an individual respondent’s role in the incident. (In a separate paper, currently underway, we will further consider the outcomes of violence.)

### Data analysis

As appropriate, we used chi-square tests or exact tests to make comparisons among the participants across the interventions. Differences in weekly reported income were examined at baseline between groups by means of *t* tests and graphical analysis, with the Health/Control camp serving as the reference. Differences in average weekly income between baseline and each time point were also assessed and tested by means of *t* tests and across groups by means of the analysis of variance (ANOVA). The linear trend in income was estimated by regressing income as a continuous variable across time points from baseline to 6 months postintervention, with the correlation between multiple measures per person accounted for by using generalized estimating equations (GEE) to correct standard errors. The analysis was unadjusted, as most differences across groups were considered relatively minor and the sample size was limited. Spaghetti plots were used in graphical analysis to assess individual patterns of income change over the duration of the study.

## Results

The four camps enrolled in the study yielded a sample of 57 participants. Although we had told the camp leaders that 15 men aged 20–35 years from each camp should come to the first session, the group that attended did not exactly meet those specifications. The Health/Control intervention camp had only 12 in attendance. One of the 57 attendees was over age 35, and 24 were under age 20 (although 16 of them were at least 20 by the end of the intervention). Two attendees were women. Because the attendees who did not meet the age and gender criteria met the other criterion of this real-life study, being unemployed or underemployed, and because we wished to avoid antagonism or tension, we accepted those who did not meet certain criteria into the study.

Four of the 57 participants were lost after enrollment and baseline data collection: one (in the Health/Control intervention) was killed after he was caught stealing, and three others returned to their homes outside the Dar es Salaam region. There were complete interview data for 35 participants. The remainder reported missing interviews because they were traveling, “busy,” or working. Cumulatively, 195 interviews took place, which constituted 90% of 216 possible interviews (not counting those that would have been conducted with participants who migrated or died).

### Characteristics of the sample

As shown in Table 2, at baseline, the ages of the participants ranged from 15 to 37 years, with a median age of 20 and a mean age of 22. The mean age was higher in the Entrepreneurship camp than in the other study camps (26 years, compared to 19–21 years in the other camps, *p* < .0001). More than 92% of the participants owned a cell phone at any given time during the study.

**Table 2:** Demographic characteristics and reported income (in Tanzanian shillings) of the study sample

Characteristic		Study camps				
	Overall (*N* = 57)	All interventions (*n* = 15)	Entrepreneurship (*n* = 15)	Beekeeping (*n* = 15)	Health/Control (*n* = 12)	*p* value*
	*n* (%)	*n* (%)	*n* (%)	*n* (%)	*n* (%)	
Educational attainment						.41
Primary	24 (42.1)	5 (33.3)	5 (33.3)	8 (53.3)	6 (50.0)	
Secondary	25 (43.9)	8 (53.3)	5 (33.3)	6 (40.0)	6 (50.0)	
Vocational training	5 (8.8)	2 (13.3)	2 (13.3)	1 (6.7)	0 (0.0)	
Diploma	3 (5.3)	0 (0.0)	3 (20.0)	0 (0.0)	0 (0.0)	
Occupation						< .01
Dependent	5 (8.8)	4 (26.7)	1 (6.7)	0 (0.0)	0 (0.0)	
Unskilled laborer	25 (43.9)	8 (53.3)	6 (40.0)	10 (66.7)	1 (8.3)	
Driver	9 (15.8)	1 (6.7)	0 (0.0)	2 (13.3)	6 (50.0)	
Semiskilled	9 (15.8)	1 (6.7)	4 (26.7)	1 (6.7)	3 (25.0)	
Microbusiness	9 (15.8)	1 (6.7)	4 (26.7)	2 (13.3)	2 (16.7)	
Has dependents						.08
No	22 (38.6)	10 (66.7)	4 (26.7)	5 (33.3)	3 (25.0)	
Yes	35 (61.4)	5 (33.3)	11 (73.3)	10 (66.7)	9 (75.0)	
Role in violence						.17
Victim	3 (5.3)	1 (6.7)	1 (6.7)	1 (6.7)	1 (8.3)	
Participant	14 (24.6)	5 (33.3)	3 (20.0)	2 (13.3)	4 (33.3)	
Witness	15 (26.3)	6 (40.0)	4 (26.7)	1 (6.7)	4 (33.3)	
None reported	25 (43.9)	3 (20.0)	7 (46.7)	11 (73.3)	3 (25.0)	
	*Mdn* (*IQR*)	*Mdn* (*IQR*)	*Mdn* (*IQR*)	*Mdn* (*IQR*)	*Mdn* (*IQR*)	
Age (years)	20 (18–24)	18 (18–19)	25 (22–32)	22 (18–25)	18 (17.5–22)	< .01
Daily income	5000 (1750–10,000)	5000 (1500–7000)	5000 (0–10,000)	5000 (2000–10,000)	5000 (2000–10,000)	.08
Weekly income	21,000 (5000–50,000)	20,000 (4500–45,000)	20,000 (10,000–50,000)	18,000 (0–30,000)	40,000 (14,000–100,000)	.02
Asset index	7 (6–7)	6.0 (5–7)	6.0 (6–7)	7.0 (7–8)	6.0 (5–7.5)	.17

*Based on Fisher’s exact test or ANOVA. *Notes. TzS* Tanzanian shilling, *IQR* interquartile range

There were no meaningful differences among the camps in the distribution of educational attainment, number of dependents, daily income, assets, or self-reported experiences of violence as perpetrators, victims, or witnesses. Almost half, 43.9% (25), of the participants reported having no experiences with violence in the last year. Differences appeared in age (*p* < .01), occupation (*p* < .01), and weekly income (*p* < .02). The mean age and *IQR* of the Entrepreneurship camp were greater than those of the other camps, although the lower end of the *IQR* for Entrepreneurship overlapped with the *IQR*s of all the other camps except All. In terms of occupation, some of the camps focused on only one type of activity. For example, the Health/Control camp focused on motorcycles as mechanics, drivers, and apprentices. The All camp was composed of athletes (football, boxing, and traditional dancing). Most commonly, study participants earned their living as unskilled day laborers, working at construction sites or carrying loads. Others had microbusinesses selling merchandise such as used clothes and marijuana. However, all participants had occupations that were temporary and part of the informal economy. Microbusinesses tended to lack a permanent site or any employees. Only two of the drivers owned their motorcycle. At baseline, mean weekly incomes were similar across all camps, TzS 20,583 (US$9.62) to 28,300 (US$13.22), except for the Health/Control camp, in which incomes were more than double, TzS 62,333 (US$29.13). Only 5% (3) of all participants earned enough to meet Tanzania’s minimum wage of TzS 300,000 (US$135) per month.

### Suitability of the curriculum for at-risk youth

Sessions were delivered as planned except that the time between the introductory session, in April 2015, and the final session, in July 2016, was 7 months longer than planned. The main delay occurred around the time of the national elections, when people were advised to be cautious of group activities. A second delay occurred waiting until the honey harvest season for the final session.

**Fig. 1:**
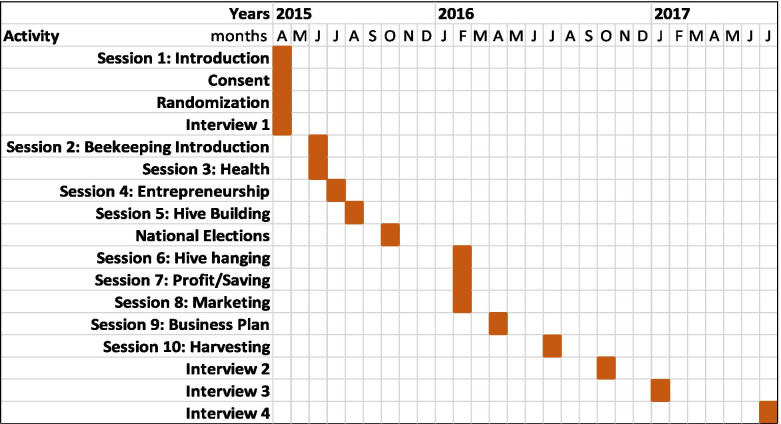
Activity timeline for Entrepreneurship, Beekeeping, and Health pilot study

As shown in Fig. 1, the time between the collection of baseline data and the final interviews in July 2017 was 2.25 years, with the intervention period lasting 1 year. With the exception of absences because of emigration or death, participants attended 90% of the training sessions.

### Quantifying bee product production after 1 year

With support from the project, the participants built six top-bar hives to specifications and two traditional hives under guidance. (Top-bar hives are so called because of the frames from which the bees suspend their wax comb.) In much of Tanzania, wild bees would be expected to move in within a few weeks. However, the government forest in which the participants hung the hives had been badly degraded by overharvesting of native timber trees and replanting with alien species. In addition, the area around the forest was cultivated by farmers who made heavy use of pesticides. After 7 months, bees had still not moved in. The final session, harvesting, was conducted with different hives. No participant who took part in the beekeeping sessions gained monetarily from the intervention itself.

### Income generation potential of four interventions after 3, 6, and 12 months

Overall, the participants’ weekly incomes increased an average of 33%. Estimates from regression analysis indicated that while the Health/Control camp experienced loss, the three intervention camps experienced income gains over the study period. The largest gain at every assessment was made by the Entrepreneurship camp (*p* = .03) which was the only camp which experienced both the Restless Development and *Ruka Juu!* training. The Entrepreneurship camp increased its weekly earnings 146%, from TzS 28,300 (US$13.22) to TzS 69,500 (US$32.48), an amount slightly less than the legal minimum wage. The Beekeeping and All camps experienced average increases of 44% and 50%, respectively. The Health/Control camp experienced a loss of 37% in its weekly earnings

The participants in the Entrepreneurship camp had diverse occupations in the informal economy. One was an electrician, some were housepainters, one was a street-level real estate agent, and others sold used clothes, children’s shoes, T-shirts, and cell phones.

A positive impact for each intervention was shown (see Table 3). From the linear regression analysis, the estimated average change in weekly income at 6 months was TzS 5249 for the Beekeeping camp (*p* = .17); the *t* test showed a real change over the study period of TzS 10,667, with a *p* value of .3453. The All intervention camp only experienced an estimated average increase of TzS 3517 (*p* = .22) at 6 months; the observed *t* test mean change for the whole study period was TzS 12,192, with a *p* value of .2175. Regression estimates for the Entrepreneurship camp showed an average income change at 6 months of TzS 14,342 (*p* = .01); the *t* test showed an observed mean change of TzS 43,292 (*p* = .0748). In a model adjusted for age to account for mean age differences between groups, inferences were unchanged.

**Table 3:** Estimated changes in weekly income from linear regression analysis, with the correlation between multiple measures per person accounted for by using generalized estimating equations

Intervention category	Estimated baseline weekly income (TzS)	Estimated baseline weekly income (TzS) *95% CI*		*p* value^1^	Estimated 6-month change in weekly income (TzS)	Estimated 6-month change in weekly income (TzS) *95% CI*		*p* value^1^
Health/Control (reference)	64,079	30,402	97,755		−6281	−18,092	5530	
All	23,065	11,484	34,646	.023	3517	−2077	9113	.217
Beekeeping	25,696	8036	43,357	.042	5249	1521	8978	.165
Entrepreneurship	29,961	18,031	41,892	.059	14,342	−189	28,873	.010

^1^*p* value for the hypothesis test as compared to the reference group

**Fig. 2:**
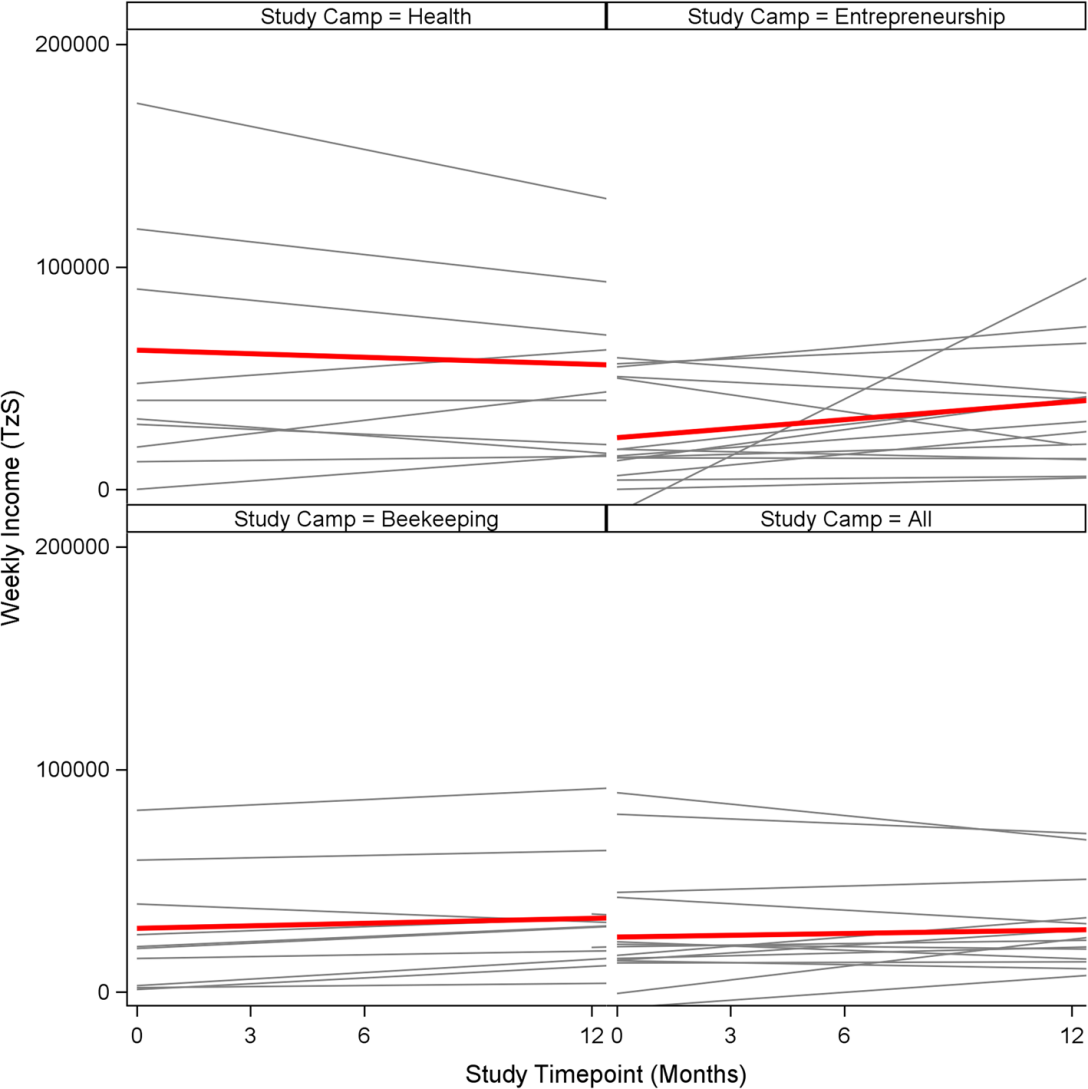
Income trajectory plots for each camp, showing individual linear change, over time, by weekly earnings. *Note*. The gray lines represent individual linear trajectories, while the red lines are the average in the linear change across the three study time points

The plots shown in Fig. 2 illustrate the weekly income linear trajectories for individuals and overall across camps and over time, from baseline through interventions through postintervention periods to a total of 2.25 years later, when the final interview was completed. The Health/Control camp showed a decrease in the reported weekly income. The three intervention camps all showed increases in weekly income, the gain being most pronounced in the Entrepreneurship camp (which had a double dose of entrepreneurship training).

A few individuals had more extreme changes. In the Entrepreneurship camp, a 22-year-old man with a primary education who was digging ditches at baseline (earnings of TzS 15,000, about US$7 in the previous week) experienced a dramatic positive change. He followed through with a business plan to print and sell T-shirts that he developed as part of the two entrepreneurship interventions of which he was part. His earnings increased 18-fold, to TzS 270,000 (about US$126) in the last two data collection times. While his experience was an outlier, all participants but one in the Entrepreneurship camp increased their incomes over the study period.

### Parameters for calculating future sample size

Our results provide preliminary data for the calculation of a sample size for the primary outcome. Based on the data from this pilot trial using results from the Beekeeping camp for the expected gain in income, and assuming a similar design with baseline preintervention measurement followed by three postintervention assessments, we estimate that we would need 37 to 72 participants per study arm to see the expected change in weekly income over the study period, depending on the method used for sample size estimation. We assumed an alpha level of .05 and a power of 90%. We also assumed a correlation between repeated measures of income of .6. Taking the most conservative approach to answering objective 5, each study arm would include 72 participants, for a total sample across four study arms of 288 participants. Extending this estimate to the cluster randomized trial context and using formulas from Hemming et al. [13], we get an optimal number of clusters (i.e., camps) per arm of 9, using a coefficient of variation (CV) of cluster size of 0.18, an intraclass correlation (ICC) across clusters of .04, and a mean cluster size of 14. The CV, ICC, and mean cluster size are all empirical estimates based on the pilot study data.

## Discussion

The key findings of the present study are as follows: the target groups can be found at the *vijiweni*, and the participants were willing to stay engaged over time; the training format was acceptable; bee product production is dependent on forces beyond the beekeepers’ control; the Entrepreneurship training has potential for income generation; the required preliminary data for the calculation of a sample size for an intervention trial has been obtained. The discussion that follows is in two parts: interpreting the results and improving the intervention in a future trial.

### Interpreting the results

#### Suitability of camps as recruitment sites

The young men found at *vijiweni* camps [39] are of the same demographic as our target group, which is characterized by a high rate of death by homicide, and whose members are themselves perpetrators of many other types of lesser crimes, such as picking pockets [29]. Those most at risk, ages 20–35 years, can be reached through these camps. We also found that a slightly younger age group (15–19 years) was well represented at the camps. Interventions such as ours are appropriate for them as well, although with less experience they may need extra support. Importantly for intervention potential, these camps appear to influence normative beliefs and risk behaviors [25] and can serve as potential points of economic stabilization for individuals [3].

#### Suitability of the curriculum

After our 6-day entrepreneurship training, the participants noted improved entrepreneurial skills and improvements in their customer care and financial management skills [17]. Since the participants were developing their own microbusinesses without monetary input from the project, it can be supposed that their improved income was a result of the training. Changes in the participants’ behaviors, attitudes, and lifestyle practices led to improved health and increased recognition and respect in their communities. Personal and social capital were enhanced [17].

Not all differences in weekly income should be attributed solely to the few training sessions. That the Beekeeping camp increased its earnings cannot be attributed to profiting from beekeeping sessions directly. Iseselo et al. ([17], p.15) suggested that it is possible that “the special attention and extra support such programs provided for youth can lead to positive outcomes … regardless of the specific design features of a program.” It is possible that participation in the program, rather than the content of the individual sessions, inspired the participants to make lifestyle changes that led to higher weekly incomes.

The Entrepreneurship camp gained the most in weekly earnings. Its members were slightly older, but our analysis did not show a significant effect of age. The participants in this camp were slightly more experienced in business (*p* ≤ .01) and were the only ones with diploma education. Balvanz et al. [3] also found that men with secondary education and with more experience in business were able to repay loans more successfully than those who were younger, with less business experience. However, the success of the Entrepreneurship camp in increasing income can probably be attributed to the double dose of training its members received from our intervention and, serendipitously, from ILO-trained instructors working for the Restless Development NGO. The latter curriculum was closer to what the respondents said they wanted during project evaluation, in that it was tailored to their education and employment levels, and took less time [17]. Overall, although speculative due to limitations in the study design, the men in the Entrepreneurship camp seem to have been able to apply the content of the general entrepreneurship sessions to their different self-chosen occupations individually, with some success.

#### Beekeeping potential

Beekeeping interventions have become popular because bees are essential to biodiversity and protecting them fosters the sustainability of the environment [32]. Moreover, through entrepreneurship, beekeepers can increase their revenues and enrich their social capital. However, beekeeping has substantial costs for start-up (hives), harvesting (beekeeper suits, ropes, smokers, buckets, containers), and packaging (jars, tops, labels, storage space) and is dependent on a safe, pesticide-free site with flowering plants nearby [14]. Harvesting is seasonal. The success of the harvest depends on many factors over which the beekeeper has little control—for example, bee behavior, weather patterns, and whether pesticides are used in the immediate area. Beekeeping has excellent potential as a source of supplemental income for beekeepers living close to their hives placed in a safe, nontoxic environment [5, 31].

#### Income generation potential

The project interventions showed three levels of response. The Health/Control camp lost more than one-third of its weekly income, a decrease in line with a contraction at the time in the national economy. All three intervention camps gained income; however, only the Entrepreneurship camp appeared to experience statistically significant change. While Beekeeping and All gained around 50% in weekly income, the Entrepreneurship camp, which experienced two doses of entrepreneurship training, gained 146%.

Youth in the Africa Region generally have strongly positive attitudes toward entrepreneurship and view business as a more common source of employment than young people in other regions of the world. For instance, about 50% of the informal trainees interviewed for an ILO study in Malawi claimed that they wanted to start their own business after informal training. According to the same report, in Tanzania, 80% of informal trainees started their own company [8].

#### Associations between macroeconomic events and changes in participants’ income

An important confounder of the present pilot study was that it occurred in the same year as competitive and unpredictable national elections. Political campaigns began in August 2015 for elections that were held the following October. During the study period, the value of the Tanzanian shilling decreased by 20%. Almost all of this loss occurred in the 6-month interval between collection of the baseline data and finalization of the results of the national elections, after which the shilling stabilized, though it did not recover. The contraction of the Tanzanian macroeconomy was mirrored by the performance of the Health/Control camp. This camp was organized around commercial motorcycle transport, which is a poor person’s “luxury” service. Most people who had been relying on commercial motorcyclists for transport opted for less expensive options such as taking the bus or walking when the money supply tightened.

Against the tide, incomes rose for all three intervention camps. The camp that showed the greatest income generation potential was the one that received a double dose of entrepreneurship training—the Entrepreneurship camp.

### Improving a future trial

Our finding that there were no meaningful differences between camps in self-reported experiences of violence at baseline illustrates a commonality. Considering the differences in income, it will be important to examine differences in experiences of violence over time. An unexpected finding was that the young men in the study considered being a witness to violence to be a form of reportable violence. This finding aligns with that of Wilkins et al. [37], who demonstrated that many forms of violence share common risk and protective factors, as well as common consequences. These common risk factors are income inequality, diminished economic opportunities, high unemployment rates and economic stress, low educational achievement, and having been a witness to violence. Protective factors include family and community support and connectedness. In a future trial, we will need to examine risk and protective factors, including being a witness to violence.

The proposed intervention trial will address research gaps by examining the effects of training and savings upon income experienced by those most at risk for violence—young men. We will also examine the impact of entrepreneurial training on all types of violence: not just the perpetration of violence against an intimate partner (as explored by Maman et al. [21]), but perpetration, victimization and witnessing of interpersonal (family and community) violence, and collective violence.

#### Entrepreneurship training

Results of entrepreneurship programs vary widely [23]. Among the areas where such programs have been shown to work is Africa. For example, as part of a randomized experiment in Ghana, basic-level management training was found to improve business practices and performance [22].

In a future trial, our training will be tailored to our target group in keeping with the insights of Iseselo et al. [17] and Smith and Perks [33]. We will adopt the more compact training of Restless Development, and note the importance of business experience, with inexperienced members receiving extra coaching.

Since almost all the study participants had cell phones, another approach that could be appropriately investigated would be to develop a standard teaching approach and compare it with an online platform that the target group could access on their phones. Issues that need to be addressed by both pedagogical approaches are compatibility (in terms of timing and operating systems), Internet connectivity, human contact with the teacher, motivation, and cost.

It is stressed in the literature that appropriate training [38] and access to such training [34] are important. Because they have dependents whose needs cannot be deferred, most workers find that full-time formal education is not an option. The timing of intervention needs to be adjusted to suit the participants’ conditions [28]. Informal workers also need to be subsidized for at least their lost earnings [12]. In our intervention, tactics such as conducting sessions on Saturdays and reimbursing the participants for any lost daily wages helped us achieve high attendance rates.

#### Start-up capital and microfinance

Across industries, geographic regions, and corporate cultural bounds, entrepreneurs report that their main constraints are a lack of entrepreneurial skills and insufficient start-up capital [6, 7, 17]. Generally, youth are not trusted because of their lack of experience and/or collateral [8, 30].

A youth entrepreneurship report revealed that in all regions of the world, 70% or more finance their enterprises by drawing upon their personal savings or by receiving assistance from family and friends [8]. The proportion is highest in the Africa Region, with 77.7% of young people using such funding sources. The ILO school-to-work transition surveys provide detailed information at the country level. In Malawi, 27% of young entrepreneurs said that no financing was needed to start their business. For about 42%, personal savings were the source of funding, and for 24%, friends and family provided financial resources. Four percent of young entrepreneurs received funding from formal financial institutions and the remainder received remittances [26]. The situation was very similar in Zambia [6, 7]. For young entrepreneurs in Swaziland, support for training was more effective than subsidies in stimulating productive start-ups [6]. The research appears to indicate that limited access to finance for young entrepreneurs should be seen more as a feature of doing business than as a constraint.

A helpful approach to the problem of lack of capital may be to teach the target group about how to manage their funds through participation in savings groups such as Village Savings and Loan Associations (VSLAs). The advantage of such groups is that the capital comes from the savings of the group members rather than outside sources, and it is possible to start with very small amounts. Each group has a constitution and meets on a regular basis to buy shares, distribute loans, and contribute to an emergency fund, with a trainer often initially facilitating these activities (see, e.g., [1]). Rather than go to a financial institution, the interest can be taken out as dividends by the members or added as capital to the revolving fund.

### Limitations

By its nature, a pilot trial is not designed to definitively test the intervention; rather, the purpose is to establish feasibility and the potential for the intervention to be effective. However, statistical testing was done in the present study to tentatively test the broad effectiveness of the interventions and to discover important associations in the study variables. There were 54 individuals with weekly income data so little data were missing for baseline analyses. For the longitudinal analysis, there were 144 observations used which corresponds to approximately 2.5 observations per person. We used generalized estimating equations (GEE) to account for the repeated measures per person which assumes missing completely at random. If the missingness was differential, this could cause a bias.

The study’s main limitation was that participation in each arm was relatively small, yielding overall small sample sizes. The small sample size was based on budgetary constraints, and on the fact that our study was largely concerned with testing the feasibility of the interventions. Using four camps in order to collect data on the acceptability, feasibility, and protocol success was sufficient to meet the study objectives. However, having only one camp per intervention meant the study was not truly randomized; therefore, the possibility exists that the observed differences between camps in income trajectories were due to uncontrolled confounding factors. This problem will be minimized in a future trial with true cluster randomization of the interventions. Furthermore, there was contamination, in that study participants shared detailed information with each other about the sessions. They met each other at the Introduction and Health sessions in which they all participated. To avoid this contamination in a trial, camps need to be geographically separated, and training should be conducted separately, at different times. It should be noted that 8–15 members are the group size preferred by the Tanzanian government for entrepreneurship training interventions, including beekeeping exercises, and our sample size calculations, based on our pilot study results, likewise indicate that this is the optimal size.

## Conclusion

The present pilot study has demonstrated the feasibility and potential for effectiveness of a short training program on entrepreneurship skills for underemployed, poorly educated young men in an urban setting in Tanzania. We have set the stage to test our updated hypothesis: Increased earnings following a 5–7-day intervention on entrepreneurship and/or microfinance will lead to decreased violence. We are ready to conduct an intervention trial with four arms: (a) Health/Control, (b) Entrepreneurship training in person, (c) Entrepreneurship training via cell phone, and (d) Entrepreneurship and VSLA training, which will enable us to evaluate the extent of improvements in entrepreneurial skills and the role of VSLAs, with a view to learning how these interventions can result in increased income and reduced exposure to violence.

## Data Availability

The data sets analyzed during the present study are available from the corresponding author on reasonable request. The data sets and materials will be deposited in the MUHAS Data Repository and at ClinicalTrials.gov.

## Abbreviations

ANOVA: Analysis of variance
CV: Coefficient of variation
GEE: Generalized estimating equations
HIV/AIDS: Human immunodeficiency virus/acquired immunodeficiency syndrome
ICC: Intraclass correlation
ILO: International Labor Organization
*IQR*: Interquartile range
MUHAS: Muhimbili University of Health and Allied Sciences
NGO: Nongovernmental organization
TzS: Tanzanian shilling
VSLA: Village Savings and Loan Association
